# Highly Asymmetric Water Permeation in Dense Laminated Membranes

**DOI:** 10.1021/acsapm.5c03886

**Published:** 2026-01-08

**Authors:** Luca Grillo, Christoph Weder

**Affiliations:** Adolphe Merkle Institute, 27211 University of Fribourg, Chemin des Verdiers 4, Fribourg 1700, Switzerland

**Keywords:** laminated membranes, dense membranes, water permeability, asymmetric permeation, moisture-induced plasticization, adhesion promoter

## Abstract

Directional permeation through dense laminated membranes is relevant for applications in various fields, including separation processes, wound care, and packaging. While theoretical models have been used to describe the asymmetric permeation in heterogeneous dense membranes, only a few systems have been experimentally explored. Here, we report dense asymmetric laminated membranes based on hydrophilic poly­(vinyl alcohol) (PVA) and hydrophobic glycol-modified poly­(ethylene terephtalate) (PETG). Modeling the system allowed us to optimize the thickness of the PVA and PETG layers. While bilayer membranes made from the two components suffered from poor interfacial adhesion and delamination, this problem is overcome by using a thin poly­sty­rene-*block*-poly­(ethylene-*ran*-butylene)-*block*-polystyrene-*graft*-maleic anhydride (SEBS-MA) adhesive layer. The maleic anhydride groups (MA) react with the hydroxyl groups present in the two polymers, and this greatly improves the adhesion between the hydrophilic and the hydrophobic layers. Membranes with optimized geometry display an asymmetry factor of up to 6.7, one of the highest values ever reported. The directional water transport is caused by the moisture-induced plasticization of the PVA layer at high relative humidity (RH), which occurs only when the PVA side of the membrane is exposed to moisture.

## Graphical abstract

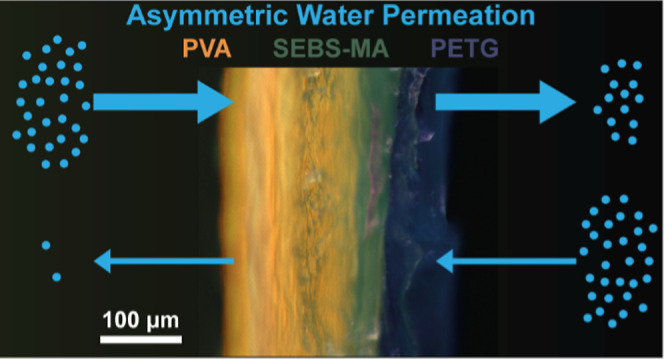

## Introduction

Mass transport through a membrane is considered asymmetric when the permeation rate of a chemical species depends on the direction of permeation.^1,2^ One of the first reports of this effect dates back to 1941, when Hurst discovered that the permeation of water through the cuticle of *Calliphora larvae* depends on the direction of transport.^3^ Hurst attributed this directional feature to the asymmetric composition of these biological membranes, which comprise an internal hydrophilic protein-chitin mix and an external hydrophobic lipid layer.^3,4^

The importance of structural heterogeneity for asymmetric permeation was highlighted in the late 1950s by Rogers and co-workers, who derived the theoretical conditions necessary for this phenomenon,^2,5–7^ and developed the first prototypes of dense laminated polymeric membranes with directional water transport properties.^2^ The first requirement for directional transport characteristics is structural heterogeneity of the membrane along the direction of transport, which can be introduced by varying the chemical composition or by tailoring physical characteristics, e.g., the degree of crystallinity or the cross-linking density.^8^ The second requirement is that at least one of the components must exhibit a permeability that varies with the vapor pressure of the permeant species.^2,5^ Of course, water vapor transport through a membrane always occurs along the gradient of water vapor partial pressure (or equivalently, chemical potential), typically from high to low vapor pressure, but in asymmetric membranes that satisfy the requirements outlined above, the transport rate depends on which membrane side faces the high-water-vapor-pressure side.

Several researchers developed mathematical models to predict and optimize the directional permeation through heterogeneous membranes, considering both graded and laminated structures.^9–14^ In one of these works, Petropoulos treated binary systems in which one component exhibits a permeability coefficient that varies with the vapor pressure of the permeant species, while the permeability of the second component is constant and concentration-independent.^13^ Depending on the mathematical correlations between the permeability and the vapor pressure, Petropoulos demonstrated that the asymmetry factor (AF), defined as the ratio of the permeability coefficients in two opposite directions of transport, can be optimized by properly tuning the parameters affecting the anisotropy, i.e., the thickness of the layers in laminated membranes or the spatial variation in composition in graded membranes.^13^ Despite these modeling efforts, few experimental studies were performed to validate such theoretical works, and the potential of asymmetric mass transport through dense heterogeneous membranes remained underexplored.^1^ This is somewhat surprising, as directional transport is a priori of interest for numerous applications, including functional clothing,^15^ separation processes,^16–18^ wound dressing,^19^ and smart packaging.^20^

To fill this gap, Kamtsikakis et al. recently developed compositionally graded nanocomposite membranes based on a hydrophobic poly­(styrene)-*block*-poly­(butadiene)-*block*-poly­(styrene) (SBS) copolymer matrix and hydrophilic cellulose nanocrystals (CNCs) as filler that showed asymmetric moisture transport.^21^ Mimicking the structural heterogeneity of olive leaf cuticles, a gradient in the CNC concentration along the transversal direction of the bioinspired membranes was created to achieve asymmetric water transport.^21^ Similar membranes made with hydrophobized CNCs offer an increased permeability of ethanol.^22^

To increase the AF of such membranes, we substituted the highly crystalline CNCs,^23^ which are impermeable and allow the water transport only along their hydrophilic surface,^21,24^ with poly­(vinyl alcohol) (PVA) nanofibers.^25^ The amorphous domains of PVA can uptake water,^26–28^ which acts as a molecular lubricant that disrupts the hydrogen-bonded PVA network and enhances the chain mobility of the polymer.^29–32^ This effect causes the water permeability (WP) to increase at high relative humidity (RH).^29,30,33–35^ Given the asymmetric distribution of the PVA nanofibers within the SBS-PVA nanocomposites, such moisture-induced plasticization occurs preferably if the PVA-rich side of the membranes is exposed to high RH, thus rendering the asymmetric water transport a switchable feature, similar to the one observed in the olive leaf cuticle.^21,25^

Based on the analysis of the experimental data using the resistance-in-series model, we investigated laminated membranes consisting of a thick PVA layer and a thin SBS layer, with the aim of maximizing the asymmetry factor attainable by this specific combination of materials.^36^ As expected, the design change led to an increase in the AF from 2.3 for the best SBS-PVA nanocomposite membrane to a value of 5.8 for a PVA-SBS bilayer membrane with optimized layer thickness.^25,36^ We concluded that the main constraint to increasing the AF further is the low WP difference between SBS and the fully water-plasticized PVA.^36^

To overcome this limitation, we set out to replace SBS, which has a WP of ca. 2.1 × 10^–14^ kg m m^–2^ s^–1^ Pa^–1^, irrespective of the RH,^21,25,36^ with a better water barrier. Based on work by Blom et al., we pivoted to glycol-modified poly­(ethylene terephtalate) (PETG), whose WP is an order of magnitude lower than that of SBS.^37^ Following the mathematical procedure described by Petropoulos,^13^ we modeled the transport through laminated PVA–PETG bilayer membranes and identified optimal thickness combinations for the PVA and PETG layers. Gratifyingly, such membranes display an asymmetry factor of 6.7, which is slightly higher than the record value of 6.5 displayed by graded poly­(ethylene-*graft*-vinyl alcohol) membranes.^5^ To ensure good adhesion between the PVA and PETG layers at high RH, i.e., under conditions where asymmetric permeation occurs, we introduced an intermediate adhesive layer consisting of polystyrene-*block*-poly­(ethylene-*ran*-butylene)-*block*-polystyrene-*graft*-maleic anhydride (SEBS-MA). The maleic anhydride (MA) residues can react with the hydroxyl groups present in both PVA and PETG,^38^ and therefore SEBS-MA greatly improves the adhesion between the hydrophilic and the hydrophobic layers.^39,40^

## Experimental Section

### Materials

Glycol-modified poly­(ethylene terephthalate) (PETG) (EASTAR 5011, *M*_n_ ∼30,000 g mol^–1^, D̵ = 1.6 measured by size exclusion chromatography) was obtained from EASTMAN. Poly­(vinyl alcohol) (PVA) (Mowiol 20–98, *M*_w_ ∼125,000 g mol^–1^), polystyrene-*block*-poly­(ethylene-*ran*-butylene)-*block*-polystyrene-*graft*-maleic anhydride (SEBS-MA), potassium chloride (KCl), potassium sulfate (K_2_SO_4_), calcium chloride (CaCl_2_) (anhydrous, granular ∼1–2 mm), indigo, rhodamine B isothiocyanate, fluorescein 5(6)-isothiocyanate, anhydrous dimethyl sulfoxide (DMSO) were supplied by Merck and were used without purification. Tetrahydrofuran (THF) (99.8%, analytical reagent grade), chloroform (99+%, for spectroscopy), and magnesium nitrate hexahydrate (Mg­(NO_3_)_2_·6H_2_O) were purchased from Thermo Fisher Scientific, and sodium chloride (NaCl) was purchased from Carl Roth. In-house deionized (DI) water was used unless otherwise specified.

### Fabrication of PVA–PETG Membranes

The PVA–PETG membranes were fabricated through lamination via compression molding of preformed films of PVA and PETG. Neat PETG films were prepared by casting solutions of PETG (1.1, 0.35, or 0.15 g) in chloroform (10 mL) into poly­(tetrafluoroethylene) (PTFE) Petri dishes (8 cm diameter). After letting the chloroform evaporate in a fume hood, the PETG films were further dried in a vacuum oven at 80 °C overnight. The films, which had a thickness of 200, 70, or 30 μm (1.1, 0.35, and 0.15 g PETG, respectively), were removed from the Petri dish and stored in a desiccator kept under vacuum. Neat PVA films were prepared by casting PVA solutions from DI water into a PTFE Petri dish (8 cm diameter). PVA solutions (40 mL) of 1.25, 2.5, and 5 wt % in DI water were used to prepare films with a thickness of 100, 200, and 400 μm, respectively. After drying in an oven at 50 °C for 2 days, PVA films were further dried in a vacuum oven at 80 °C for 24 h, and then, still in the Petri dish, transferred into a desiccator kept under vacuum to cool to room temperature (rt).

A first PVA–PETG membrane was prepared by compression molding of neat PVA (200 μm) and neat PETG (30 μm) films using a Carver press at 180 °C with a load of 4 tons for 4 min, with spacers of 250 μm. Delamination issues of this PVA–PETG prototype at high RH (vide infra) triggered a change in the preparation method, i.e., the addition of an intermediate layer of SEBS-MA as an adhesion promoter.

To prepare membranes with an SEBS-MA adhesion layer, PVA films were prepared as described above, but not removed from the Petri dish. A dilute solution of SEBS-MA (50 mg) in chloroform (5 mL) was cast directly onto the neat PVA films (still in the original Petri dish). After letting the chloroform evaporate in a fume hood, the resulting PVA/SEBS-MA membrane was further dried in a vacuum oven at 80 °C overnight and then stored in a desiccator under vacuum. The thickness of the SEBS-MA coating, determined by the difference in thickness between the PVA/SEBS-MA films and the PVA layers, was ∼15 μm. The PVA–PETG membranes were then prepared via compression molding of the preformed PVA/SEBS-MA and PETG films. The respective films were placed on top of each other (with the SEBS-MA layer facing the PETG) and hot-pressed using a Carver press at 180 °C with a load of 4 tons for 4 min. The samples are referred to as **PVA**_***x***_ **-PETG**_***y***,_ where *x* and *y* represent the thickness of the PVA and PETG layers, respectively, in μm. In order to retain the thickness of the preformed PVA/SEBS-MA and PETG layers, these components were compression molded using spacers of 250 μm to prepare the **PVA**_**200**_ **-PETG**_**70**_ and **PVA**_**200**_ **-PETG**_**30**_ membranes, while spacers of 300 and 450 μm were utilized for the **PVA**_**100**_ **-PETG**_**200**_ and **PVA**_**400**_ **-PETG**_**30**_ laminates, respectively. Although this is not reflected in the sample code, unless otherwise noted, the membranes contain the SEBS-MA adhesion layer.

### Neat PVA and PETG Reference Films

PVA reference films were prepared via solution casting of PVA in DI water (40 mL of 2.5 wt % solution) into a PTFE Petri dish (8 cm diameter). The solvent was evaporated in an oven at 50 °C for 2 days, and the resulting PVA films were further dried in a vacuum oven at 80 °C for 24 h. The films had a thickness of 200 μm and were stored in a desiccator kept under vacuum. PETG reference films were produced by compression molding pellets between PTFE sheets in a Carver press at 180 °C for 4 min with a load of 4 tons. More specifically, 0.5 g of PETG pellets were used to produce films with a thickness of 100 μm.

### Modeling of the PVA–PETG Laminated Membranes

The numerical calculations to predict the asymmetric water transport through the PVA–PETG laminated membranes were performed using MATLAB R2024B (MathWorks, USA). The MATLAB script and the raw data used for the modeling are available at 10.5281/zenodo.17055356.

### Thickness Measurements

The thickness of films and membranes was measured at a minimum of 10 random spots across the sample using a digital micrometer (IP 65, Mitutoyo). The reported values are the average ±standard deviation.

### Scanning Electron Microscopy

The morphology of the PVA–PETG laminated membranes was characterized by scanning electron microscopy (SEM) using a Tescan Mira3 LM FE with a voltage of 5 kV. The cross-section of the PVA–PETG laminated membranes was observed in samples cut with a razor blade and stored in a desiccator before imaging. The specimens were coated with a thin gold layer (4 nm) using a Cressington 208HR high-resolution sputter coater (U.K.).

### Fluorescence Microscopy

PVA was labeled with rhodamine isothiocyanate following the procedure described in Shirole et al.^41^ More specifically, PVA pellets (7 g) and the dye (0.35 mg) were dissolved in dry DMSO (70 mL), and the reaction mixture was stirred under nitrogen atmosphere at 90 °C for 3 h. The rhodamine-labeled PVA was precipitated into methanol (500 mL), filtered off, washed in methanol (300 mL), and dried at 60 °C for 24 h. SEBS-MA (50 mg) was labeled with fluorescein isothiocyanate (0.05 wt % with respect to the polymer) by mixing the two compounds in a THF solution (5 mL). PETG (150 mg) was labeled with indigo dye (0.1 wt % compared to the polymer) by mixing the two compounds in a chloroform solution (10 mL). The labeled PVA–PETG membrane was prepared following the same protocol described above for the undyed membranes, with the only difference being that SEBS-MA was cast from THF instead of chloroform to allow the complete dissolution of the fluorescein dye. Microscopy images were acquired at 20× magnification using an Olympus BX51 microscope equipped with an Olympus DP72 high-resolution camera. Samples were imaged in reflectance mode. Lateral excitation of fluorophore molecules was obtained using a UV lamp positioned at approximately 90° relative to the objective. The white balance was adjusted using a standard white diffuser prior to image acquisition.

### Tensile Testing

Measurements were conducted at ambient conditions (23 °C, typical RH ∼50%) on a Zwick/Roell Z010 tensile tester equipped with a 200 N load cell at a strain rate of 50 mm min^–1^. Dog bone-shaped samples were prepared using a die-cutter (Zwick/Roell, cutter length 38.1 mm, path length 22.25 mm, path width 4.75 mm, width 15.88 mm, and *R* = 3.2 mm). Samples tested in the dry conditions were stored in a desiccator under vacuum before testing. Moisture-conditioned samples were stored in an incubator kept at RH ∼95% (K_2_SO_4_ saturated salt solution)^42^ for 1 week before tensile testing.

### Water Permeability Measurements

Standard test methods for water vapor transmission of materials according to ASTM E96 were used to measure the water permeability of the reference PVA and PETG films and the PVA–PETG membranes.^43^ A schematic representation of the dry cup and wet cup methods used for the experiments is provided in Figure S1, while the details of the experimental procedure are described in Supporting Note 1. In the dry cup method, a steady relative humidity of the donor compartment (RH_D_) was generated using salt solutions placed in an incubator kept at *T* = 25 °C. Saturated solutions in DI water of Mg­(NO_3_)_2_ (RH_D_ = ∼55%), NaCl (RH_D_ = ∼75%), KCl (RH_D_ = ∼85%), or K_2_SO_4_ (RH_D_ = ∼95%)^42^ were used to reach RH_D_ values of 55, 70, 80, 85, 90, and 95%. The temperature and relative humidity inside the incubator were monitored using a humidity thermometer (FisherbrandTM TraceableTM humidity thermometer), and the reported values of RH_D_ are the average values of RH_D_ measured during the permeability test. The variations of the average with respect to the target values of RH_D_ are within the accuracy of the monitoring device (±4%).

### Water Uptake Measurements

Water uptake experiments were conducted at room temperature (*T* = 23 °C). The neat PVA and PETG reference films were dried and cut into square samples of 1 × 1 cm^2^, and their initial dry weight (*M*_0_) was determined before conditioning them at different relative humidities (RH ranging from 55 to 100%). The aqueous swelling was monitored gravimetrically by weighing the samples regularly until the equilibrium wet weight (*M*_∞_) was reached. The equilibrium water uptake was calculated with eq 1. The reported values are the mean and the standard deviation of *n* = 3 measurements for each film.

1 $$Equilibrium\;water\;uptake\left(\%\right)=\frac{M_{\infty }-M_{0}}{M_{0}}\times 100$$

## Results and Discussion

### Water Permeability of Neat PVA and PETG Films

In order to design laminated PVA–PETG membranes for asymmetric water permeation, we first investigated the water transport properties of neat PETG and PVA films as a function of the relative humidity of the donor compartment (RH_D_), keeping the receiver compartment at a nominal relative humidity of RH_R_ = 0%.^21^ The wet-cup method defined in the ASTM E96 standard was employed to determine the WP for RH_D_ = 100%, while the dry cup method was used to measure the WP for all other RH_D_ values (Figure S1, Supporting Note 1).^43^ The water permeabilities of PVA (reproduced from our previous work^36^) and of PETG as a function of RH_D_ are shown in Figure 1. The water permeability of PVA (WP_PVA_) highly depends on the relative humidity to which the films are exposed, increasing by almost 3 orders of magnitude as RH_D_ is changed from ∼55% (WP_PVA_ = 6.0 ± 1.0 × 10^–16^ kg m m^–2^ s^–1^ Pa^–1^) to 100% (WP_PVA_ = 2.9 ± 0.4 × 10^–13^ kg m m^–2^ s^–1^ Pa^–1^).^36^ As previously reported,^25,29,30,33–36^ this change is driven by the moisture-induced plasticization of the polymer, which in turn causes an increase in polymer chain mobility and water diffusion rate.^29,30^ On the other hand, the water permeability of the neat PETG (WP_PETG_) films is almost constant over the entire RH_D_ range investigated, with an average of WP_PETG_ = 3.5 × 10^–15^ kg m m^–2^ s^–1^ Pa^–1^, which matches the value reported by Blom et al.^37^ WP_PETG_ is an order of magnitude lower than the WP of SBS, which we utilized in our previous works.^21,25,36^ Consequently, the difference between the WPs of the two components used here, which is maximal at RH_D_ = 100% (Figure 1), increases to almost 2 orders of magnitude, with WP_PVA_ = 2.9 ± 0.4 × 10^–13^ and WP_PETG_ = 4.1 ± 0.5 × 10^–15^ kg m m^–2^ s^–1^ Pa^–1^. The two polymers also display very different equilibrium water uptake (Figure S2). While PVA shows an increase from 3.2 ± 0.5% to 53.6 ± 4.1 when RH increases from 60 to 100%, PETG reference films exhibit no significant water uptake (0.0 ± 0.4%) even at RH = 100%, indicating the good water barrier properties of this hydrophobic polymer.

**Figure 1:**
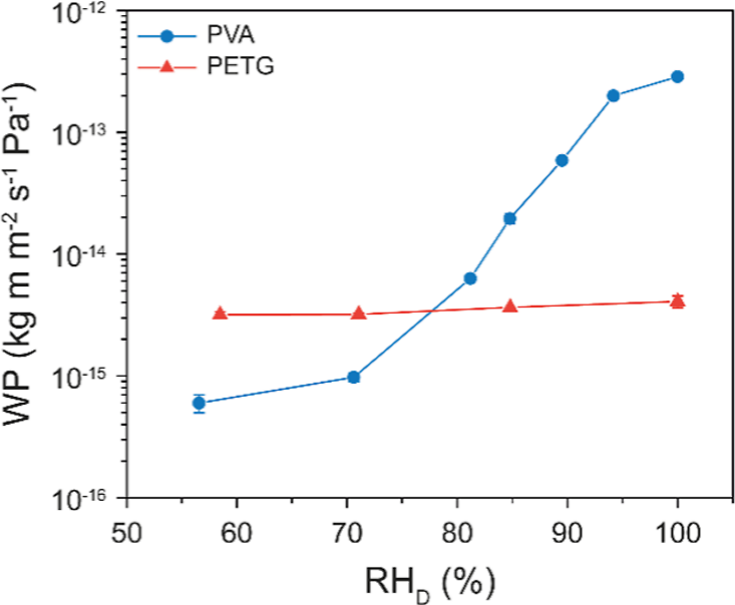
Water permeability (WP) of neat PVA and neat PETG films as a function of the relative humidity in the donor compartment (RH_D_); the relative humidity in the receiver compartment was kept constant at RH_R_ = 0%. The values reported are means of measurements on *n* = 4 different membranes, and error bars reflect standard deviations.

### Modeling of the Asymmetric Water Permeation in Laminated PVA–PETG Membranes

Having established the water transport properties of the neat PVA and neat PETG reference films, we modeled the transport through laminated PVA–PETG membranes by using the experimentally determined correlations between the water permeabilities of each material and relative humidity (Figure 1) as the primary inputs for the mathematical analysis. We followed the approach introduced by Petropoulos, who considered laminated membranes consisting of one component whose permeability varies with the vapor pressure of the penetrant species, and a second component with constant permeability.^13^ This assumption matches the materials used in this study, where the PVA has a water permeability WP_PVA_ that strongly depends on RH, while the PETG has a constant WP_PETG_ (Figure 1). In our model, the PVA–PETG membranes are represented as bilayer membranes made of a thick PVA layer and a thin PETG layer (Figure 2). Although a thin adhesion-promoting layer was used (vide infra), this element was omitted in the modeling, since this interlayer contributes only a very small fraction to the total membrane thickness (3–6%, vide infra). We thus expected (and confirmed) that its influence on the overall asymmetric permeation is minimal, and its omission greatly simplifies the modeling.

**Figure 2:**
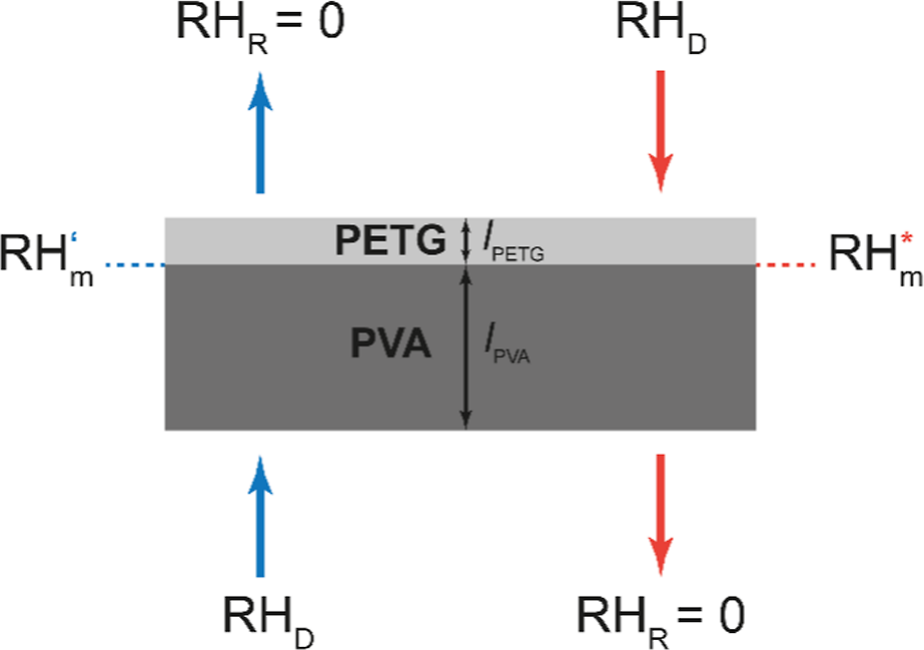
Schematic representation (not to scale) of the laminated PVA–PETG membranes and the transport directions. The blue arrow indicates the PVA → PETG transport direction, while the red arrow indicates the opposite direction, PETG → PVA. In experimental embodiments, a thin adhesion-promoting layer was used between the PETG and PVA layers, but this element was omitted in the modeling.

Key parameters for our modeling are presented in Figure 2. When the water transport occurs in the PVA → PETG direction (blue arrows), the PVA layer faces the donor compartment and is subjected to RH_D_; in this case, the relative humidity at the interface with the PETG layer is defined as RH_m_^′^. If the transport direction changes and water is transported in the PETG → PVA direction (red arrows), the PVA layer faces the receiver compartment at RH_R_ = 0. In this case, the PVA layer is exposed to water only at the interface with the PETG layer, and the intermediate relative humidity in this direction is defined as RH_m_^*^. By varying the thickness of the PVA (*l*_PVA_) and PETG (*l*_PETG_) layers, the water transport characteristics of the PVA–PETG membranes change, and, in general, RH_m_^′^ ≠ RH_m_^*^.

Knowing RH_m_^′^ and RH_m_^*^, one can express the asymmetry factor (AF_M_, where the subscript M indicates that the value is modeled) of the bilayer membranes using eq 2 (Derivation in Supporting Note 2)^13^

2 $${AF}_{M}=\frac{{RH}_{m}^{\prime }}{{RH}_{D}-{RH}_{m}^{*}}$$

As described by Petropoulos,^13^ the relative humidity values at the interface RH_m_^′^ and RH_m_^*^ can be estimated by introducing the normalized permeation rate *Ĵ*, as defined in eq 3

3 $$Ĵ=\frac{1}{l}\int_{{RH}_{R}}^{{RH}_{D}}WP\left(RH\right)dRH$$

where *l* is the overall thickness of a membrane subjected to a humidity gradient (RH_D_–RH_R_).

Under steady-state conditions, the normalized permeation rate through the laminated PVA–PETG membranes should be the same as the flow passing through the single layers of PVA and PETG. This condition is fixed by eqs 4 and 5, which are the expressions of the normalized permeation rate *Ĵ* in the PVA → PETG and PETG → PVA direction, respectively.

4 $${Ĵ}_{PVA\to PETG}=\frac{1}{l_{PVA}}\int_{{RH}_{m}^{\prime }}^{{RH}_{D}}{WP}_{PVA}\left(RH\right)dRH=\frac{1}{l_{PETG}}\int_{0}^{{RH}_{m}^{\prime }}{WP}_{PETG}$$

5 $$PETG\to PVA{Ĵ}_{PETG\to PVA}=\frac{1}{l_{PVA}}\int_{0}^{{RH}_{m}^{*}}{WP}_{PVA}\left(RH\right)dRH=\frac{1}{l_{PETG}}\int_{{RH}_{m}^{*}}^{{RH}_{D}}{WP}_{PETG}$$

More specifically, the first integral terms of eqs 4 and 5 represent the flow through the PVA layer, while the second integral terms express the flow through the PETG layer. The extremes of integration refer to the RH values to which the PVA and PETG layers are exposed according to Figure 2. Solving eqs 4 and 5 for the two unknown variables RH_m_^′^ and RH_m_^*^ allows the determination of AF_M_ using eq 2. Considering the dependence of WP_PVA_ on RH (Figure 1), the integral equations reported in eqs 4 and 5 can be solved only after evaluating WP_PVA_(RH).

As reported previously,^33,36^ the dependence of WP_PVA_ and RH assumes an S-shaped curve (Figure 1), which can be expressed by fitting the experimental WP_PVA_ data with a 4-parameter logistic function.^36^ Given the complexity of this function, the integral equations reported in eqs 4 and 5 must be solved numerically to determine RH_m_^′^ and RH_m_^*^. This was achieved by code that was run in MATLAB (See Experimental Section for details).

Considering that PVA and PETG show the highest WP difference at RH_D_ = 100% (Figure 1), we modeled AF_M_ only for this condition as it represents the state in which the contrast in WP between the two components, and thus the expected degree of asymmetric water transport, is maximized. To evaluate the best thickness combination, we varied the thickness of the PVA layer (*l*_PVA_) in the range of 100–500 μm and the thickness of the PETG layer (*l*_PETG_) in the range of 5–250 μm. Figure 3 shows how AF_M_ varies within this parameter space. The colormap indicates that all modeled thickness combinations produce asymmetric water permeation, as AF_M_ > 1. The lowest AF_M_ value of 1.8 is predicted for *l*_PVA_ = 500 μm and *l*_PETG_ = 5 μm, indicating that the asymmetric water permeation is less pronounced when the hydrophobic PETG layer is much thinner than the hydrophilic PVA layer. Indeed, for a fixed *l*_PETG_ = 5 μm, AF_M_ decreases as the thickness of the PVA layer increases (Figure 3). The opposite trend is apparent when *l*_PETG_ = 250 μm is fixed; in this case, AF_M_ grows as *l*_PVA_ increases from 100 to 500 μm (Figure 3), demonstrating that a thick hydrophobic barrier enables high AF_M_ values only when the moisture-sensitive layer is significantly thicker than the water barrier itself.

**Figure 3:**
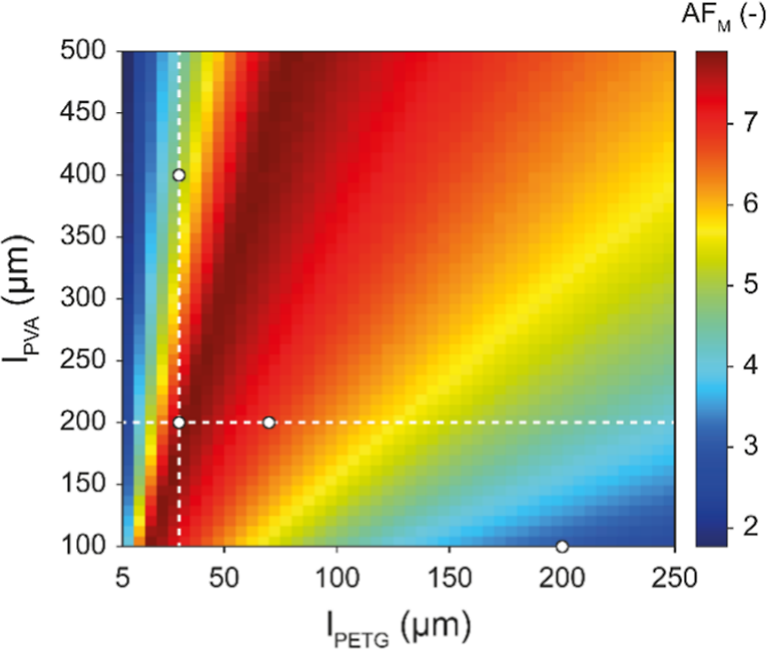
Colormap that expresses the modeled asymmetry factor (AF_M_) of laminated PVA–PETG membranes for RH_D_ = 100% as a function of the thickness of the PVA (*l*_PVA_) and PETG (*l*_PETG_) layers. The white dashed lines represent slices of the colormap at fixed *l*_PVA_ = 200 μm (horizontal line) and fixed *l*_PETG_ = 30 μm (vertical line) values, and white circles indicate the experimentally investigated compositions.

For intermediate values of *l*_PETG_, the theoretical trend of AF_M_ is more complex. For example, at *l*_PETG_ = 30 μm, AF_M_ initially increases with *l*_PVA_, reaching a maximum value of 7.9 at *l*_PVA_ ∼190 μm, after which it decreases as the thickness of the PVA layer increases (vertical dashed line in Figure 3). A similar behavior is observed when *l*_PVA_ = 200 μm is fixed: AF_M_ reaches a peak of 7.9 at *l*_PETG_ ∼30 μm and then decreases with further increases in *l*_PETG_ (horizontal dashed line in Figure 3). As indicated by the dark-red ridge in Figure 3, a maximum value of AF_M_ = 7.9 is predicted for different thickness combinations. Overall, the map shows that there is a large parameter space in which AF_M_ exceeds the value of 5.8, i.e., the maximum value that we reached by combining PVA and SBS layers.

The design of the four membranes that we experimentally investigated (white circles in Figure 3) was directly guided by the modeling results. To create a membrane with a maximum asymmetry factor, the combination of *l*_PVA_ = 200 μm and *l*_PETG_ = 30 μm was selected. Two additional configurations were selected to validate the theoretical trends of AF_M_ described for a fixed thickness of the PETG layer (*l*_PETG_ = 30 μm, *l*_PVA_ = 400 μm) and for a fixed thickness of the PVA layer (*l*_PVA_ = 200 μm, *l*_PETG_ = 70 μm). Finally, a combination of *l*_PVA_ = 100 μm and *l*_PETG_ = 200 μm was selected to assess the model’s predictive accuracy at low AF_M_ values.

### Fabrication and Morphological Characterization of Laminated PVA–PETG Membranes

The PVA–PETG membranes were prepared by laminating prefabricated PVA and PETG films whose thickness was carefully controlled to match the target values discussed above. Thus, PETG films with a target thickness of 30, 70, and 200 μm were prepared by casting chloroform solutions of the polymer and subsequent drying, while PVA films with a target thickness of 100, 200, and 400 μm were made by solution-casting from DI water. In both cases, the film thickness was controlled via the concentration of the casting solution (See Experimental Section for details).

A first series of PVA–PETG bilayer membranes was prepared by compression molding the individual films in a hot press at 180 °C. This temperature was chosen because it erases any crystalline domains that may have formed during the solution casting of PETG,^44^ and it is also below the melting temperature of PVA. Spacers were employed to maintain the thickness of the original layers. The transparent membranes thus produced were initially intact, but the two layers delaminated upon conditioning at RH ∼95% for a day (Figures S3, Supporting Video S1).

We thus explored the use of an adhesion-promoting intermediate layer. Inspired by the work of Lira et al., who demonstrated that the maleic anhydride groups present in polystyrene-*block*-poly­(ethylene-*ran*-butylene)-*block*-polystyrene-*graft*-maleic anhydride (SEBS-MA) can react with the carboxyl groups of PETG and the hydroxyl groups present in both PETG and PVA, most likely forming ester linkages (Figure S4),^38^ we speculated that using SEBS-MA as an intermediate layer would enhance the adhesion between PETG and PVA. Similar approaches have already been used to enhance the compatibility between other combinations of hydrophobic and hydrophilic polymers in multilayer films.^39,40^ Thus, PVA films of different thicknesses were coated with a ca. 15 μm-thick SEBS-MA layer that was applied by solution casting from chloroform. Laminated membranes were then produced by compression molding the SEBS-MA-coated PVA films and PETG films at 180 °C. These membranes are referred to as **PVA**_***x***_ **-PETG**_***y***_, where *x* and *y* represent the original thickness of the PVA (*l*_PVA_) and PETG layers (*l*_PETG_), respectively, in μm. The data shown in Table 1 reflect that the total membrane thickness (*l*_T_) corresponds to the sum of the thickness of the original layers, i.e., the layer thickness can be precisely tuned by selecting proper spacers during the compression molding (See Experimental Section for details).

**Table 1:** Thickness of the PVA (*l*_PVA_), SEBS-MA (*l*_SEBS‑MA_), and PETG (*l*_PETG_) Layers, and the Total Thickness (*l*_T_) of the Various **PVA**_***x***_ **-PETG**_***y***_ Membranes Studied

membrane	*l*_PVA_ [μm]^a^	*l*_SEBS‑MA_ [μm]^b^	*l*_PETG_ [μm]^a^	*l*_T_ [μm]^a^
**PVA**_**100**_ **-PETG**_**200**_	100 ± 6	14 ± 1	213 ± 6	318 ± 6
**PVA**_**200**_ **-PETG**_**70**_	200 ± 9	14 ± 1	68 ± 6	265 ± 12
**PVA**_**200**_ **-PETG**_**30**_	207 ± 12	15 ± 1	31 ± 4	252 ± 8
**PVA**_**400**_ **-PETG**_**30**_	408 ± 22	12 ± 1	32 ± 3	453 ± 18

a Experimentally determined thickness of the as-prepared PVA and PETG films and the assembled membranes.

b Back-calculated from the experimentally determined values.

To investigate the morphology of the multilayer **PVA**_***x***_ **-PETG**_***y***_ membranes, their cross sections were imaged by scanning electron microscopy (SEM). The SEM images clearly show the presence of the SEBS-MA intermediate layer and reveal intimate interfaces between the latter and the PVA and PETG layers (Figures 4, S5). The fluorescence microscopy image of the cross-section of a **PVA**_**200**_ **-PETG**_**30**_ membrane, in which each layer was labeled with a different fluorescent dye, confirms the SEM images and suggests the absence of layer mixing, indicating that the materials maintain their integrity after the compression molding (Figure S6).

**Figure 4:**
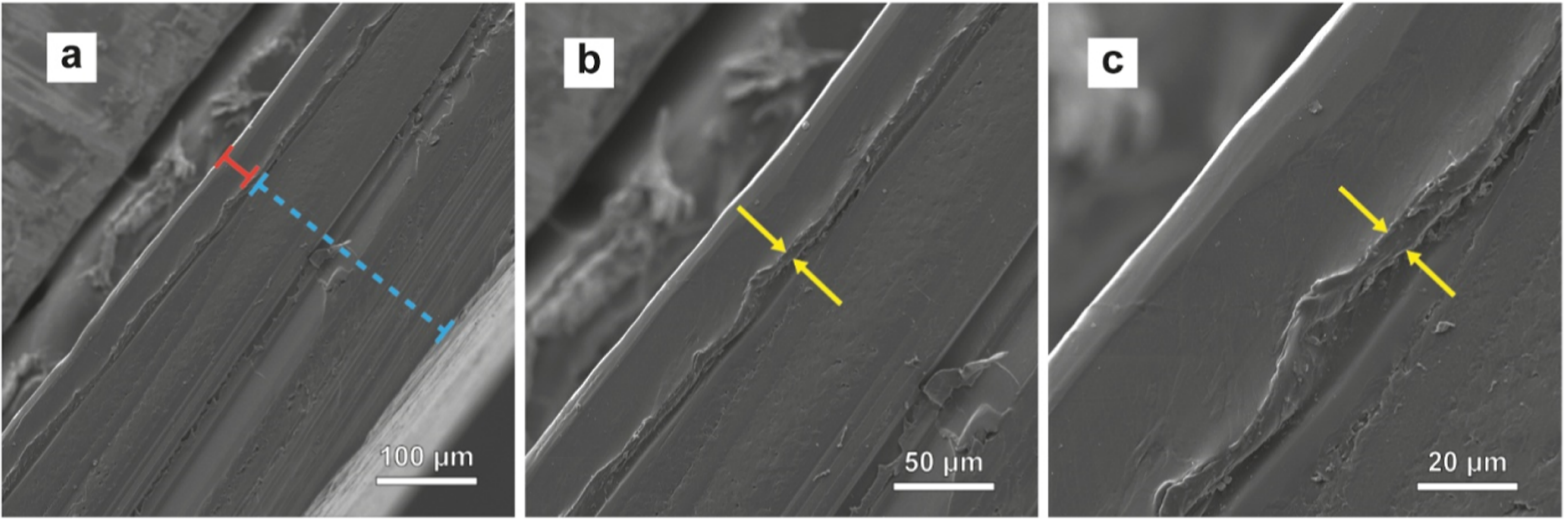
SEM images of the cross-section of **PVA**_**200**_ **-PETG**_**30**_ laminated membranes at (a) 1000×, (b) 2000×, and (c) 5000× magnifications. The dashed blue lines indicate the PVA layer, the solid red lines indicate the PETG layer, and the yellow arrows indicate the SEBS-MA intermediate layer.

### Mechanical Characterization

The mechanical properties of the PETG and PVA reference films and the **PVA**_**200**_ **-PETG**_**30**_ membranes, which were investigated as representative of the series, were assessed by tensile testing (Figure 5). The stress–strain curves of the dry samples recorded at ambient temperature (Figure 5a), i.e., far below the glass transition temperature (*T*_g_) of the semicrystalline PVA and the amorphous PETG, reflect the well-known behaviors of these materials. The stress–strain curve of PVA films shows an elastic low-strain regime with a Young’s modulus (*E*) of 4.3 GPa, the onset of plastic deformation at a strain of ca. 4%, a high tensile strength (σ) of 95 MPa, and a moderate strain at break (ε_B_) of 16% (Table 2). The glassy PETG is less stiff (*E* = 1.7 GPa), less strong (σ = 40 MPa), and less ductile, showing brittle failure at ε_B_ = 3.0%. The stress–strain curve of the dry **PVA**_**200**_ **-PETG**_**30**_ membrane closely traces that of the PVA reference film, but *E* and ε_B_ are slightly lower, reflecting that the presence of the PETG makes the assembly more prone to the brittle failure observed in the reference film of PETG. However, the stress–strain curves of the multilayer membranes do not show a discontinuity that would indicate fracture of the PETG layer or delamination, and no such events were visually detected. Under dry conditions, the **PVA**_**200**_ **-PETG**_**30**_ membrane breaks uniformly (Figure S7), without any indications of a fracture of the PETG layer, as confirmed by the SEM images of the PETG layer after the tensile test, which appears deformed but not fractured (Figure S8a,b).

**Figure 5:**
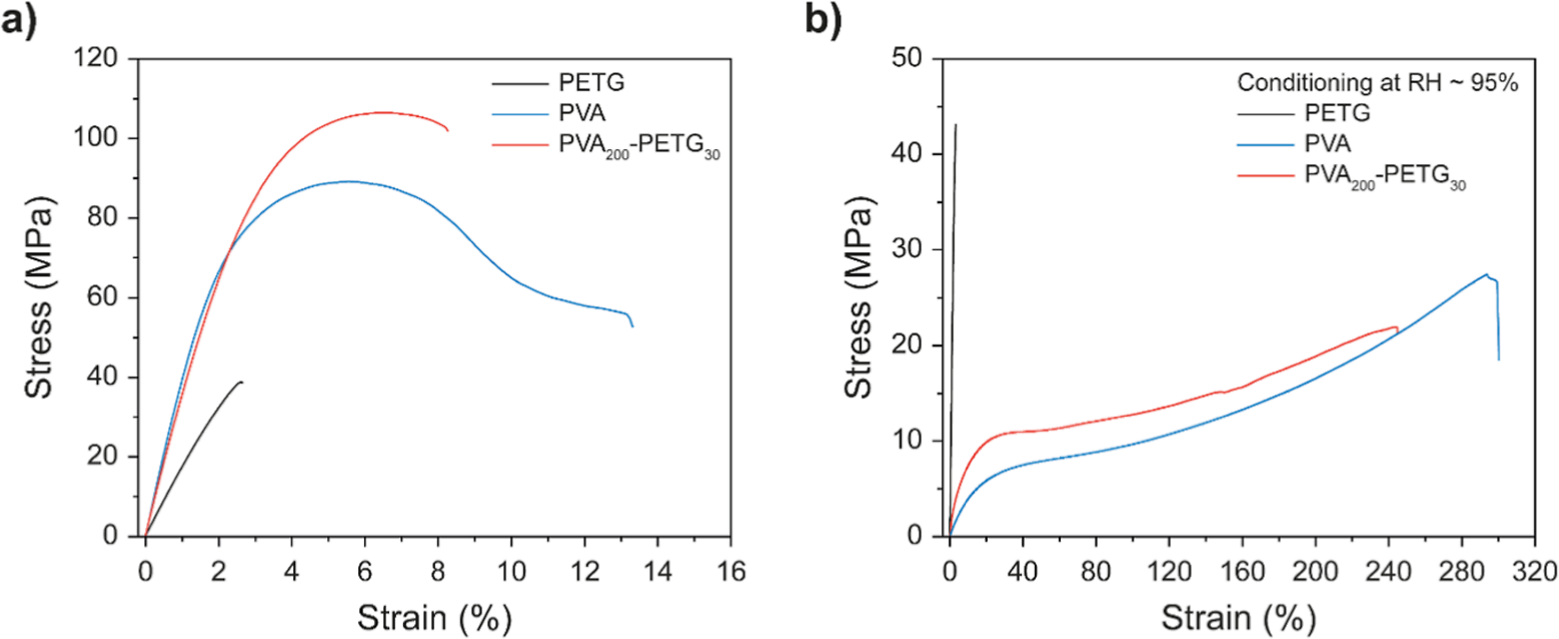
Representative stress–strain curves of the neat PETG and PVA reference films and **PVA**_**200**_ **-PETG**_**30**_ membranes under (a) dry conditions and (b) after conditioning at RH ∼95% for 1 week.

**Table 2:** Mechanical Properties of the Neat PETG and PVA Reference Films and **PVA**_**200**_ **-PETG**_**30**_ Membranes Determined by Tensile Tests^a^

sample code	Young’s Modulus *E* [MPa]	elongation at break ε_B_ [ %]	tensile strength σ [MPa]
**PETG**	1725 ± 192	3.0 ± 0.4	40 ± 3
**PETG (95%**RH)	1714 ± 68	3.2 ± 0.2	43 ± 2
**PVA**	4273 ± 330	16 ± 2	95 ± 9
**PVA (95%**RH)	62 ± 4	296 ± 16	26 ± 3
**PVA**_**200**_ **-PETG**_**30**_	3997 ± 277	9 ± 1	110 ± 5
**PVA**_**200**_ **-PETG**_**30**_ **(95%**RH)	200 ± 15	252 ± 19	22 ± 2

a All data were acquired at room temperature and represent averages of at least *n* = 3 individual measurements ±standard deviation.

As previously reported,^31,32,36^ the exposure of PVA to ∼95% RH leads to a water uptake of 23 wt % and causes the *T*_g_ to drop from 87 °C in the dry state (RH = 0%) to −23 °C.^36^ This water-induced plasticization significantly affects the mechanical properties (Figure 5b, Table 2).^32^ *E* drops by almost 2 orders of magnitude to 62 MPa, σ is reduced to 26 MPa, and a massive increase in ε_B_ to ca. 300% is observed. By contrast, the mechanical characteristics of the PETG reference film (*T*_g_ = 83 °C) exhibit no statistically significant changes upon exposure to ∼95% RH (Figure 5b, Table 2). Given that the majority component is PVA, the mechanical behavior of the **PVA**_**200**_ **-PETG**_**30**_ membranes after conditioning at ∼95% RH is also strongly affected by the plasticization of the PVA layer, and the properties again largely mirror those of the conditioned PVA films. However, the presence of the stiffer PETG layer causes *E* of the laminated membranes (200 MPa) to exceed that of the plasticized PVA reference.

Very much in contrast to the PVA–PETG membranes produced without the SEBS-MA intermediate layer (Figures S3, Supporting Video S1), the **PVA**_**200**_ **-PETG**_**30**_ membranes show no signs of delamination upon exposure to high relative humidity (∼95% RH, Supporting Video S2), even upon deformation (Supporting Video S3), further supporting the role of the reactive interlayer in enhancing adhesion between the hydrophilic and hydrophobic components.^39,40^ Post-mortem SEM images of the PETG side of the conditioned laminated membranes taken after tensile testing and failure at an average strain of ε_B_ = 252% show the presence of microscopic fractures in the rigid PETG layer (Figure S8c,d). Thus, while the integrity of the PETG layer is locally compromised at such a high strain, which largely exceeds the failure strain of the neat PETG reference films (ε_B_ = 3.2%), macroscopic delamination in the laminated membrane is prevented. This behavior highlights the effectiveness of the SEBS-MA interlayer, which maintains strong interfacial adhesion between the PETG and PVA layers even when the PETG component has fractured.

### Asymmetric Water Permeation in Laminated PVA–PETG Membranes

After confirming the mechanical stability of the laminated **PVA**_**200**_ **-PETG**_**30**_ membranes, we investigated their water transport properties as a function of the transport direction. We recall that the model predicts AF_M_ = 7.9 for this particular combination of layer thicknesses (Figures 3, 6). The experimental asymmetry factor AF_E_ (where the subscript E is used to distinguish the experimentally determined value from the modeled value AF_M_) was determined by employing the wet-cup method with RH_R_ = 0% and RH_D_ = 100% and measuring the water permeability in the directions from the PVA layer to the PETG layer (WP_PVA→PETG_) and in the opposite direction (WP_PETG→PVA_). AF_E_ was then calculated by applying eq 6

6 $${AF}_{E}=\frac{WP_{PVA\to PETG}}{{WP}_{PETG\to PVA}}$$

**Figure 6:**
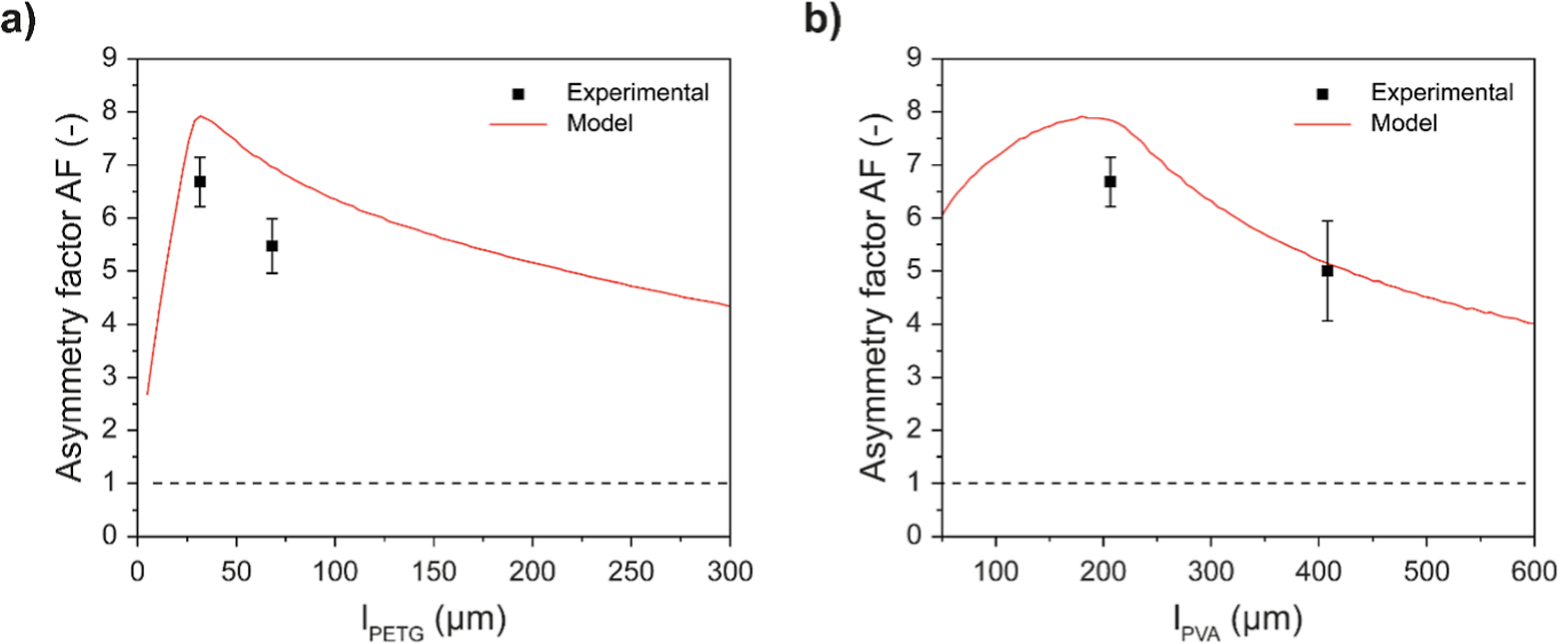
Comparison between the asymmetry factor of laminated **PVA**_***x***_ **-PETG**_***y***_ membranes predicted by the model (AF_M_) and the experimentally determined values (AF_E_) at RH_D_ = 100% and RH_R_ = 0% for different thickness combinations. (a) Effect of the thickness of the PETG layer *l*_PETG_ on AF with fixed PVA thickness *l*_PVA_ = 200 μm. (b) Effect of the thickness of the PVA layer *l*_PVA_ on AF with fixed *l*_PETG_ = 30 μm. The experimental data were measured with the wet cup method and are the mean ± s.d. of *n* = 3 membranes.

This analysis afforded an AF_E_ = 6.7 ± 0.5, which is only slightly lower than the predicted AF_M_ and exceeds the highest value that we previously measured for PVA-SBS bilayer membranes with similar dimensions (AF_E_ = 5.8).^36^ The AF_E_ is also comparable to the record value set by Rogers’ graded poly­(ethylene-*graft*-vinyl alcohol) membranes, with an AF = 6.5.^5^ We emphasize that the laminated **PVA**_***x***_ **-PETG**_***y***_ membranes reported here are much easier to prepare than such graded membranes.^5^

To evaluate the accuracy of the model’s predictions further, we experimentally explored three other **PVA**_***x***_ **-PETG**_***y***_ membranes with other layer thickness combinations. Figure 6 shows two AF_M_ traces that represent slices from the 2D colormap presented in Figure 3 at fixed *l*_PVA_ (200 μm, Figure 6a) and fixed *l*_PETG_ (30 μm, Figure 6b), as well as the experimental AF_E_ data of the **PVA**_**200**_ **-PETG**_**30**_, **PVA**_**200**_ **-PETG**_**70**_, and **PVA**_**400**_ **-PETG**_**30**_ membranes. The AF_M_ traces presented in Figure 6 show that if the thickness of one layer is fixed, there exists a unique value of the second layer’s thickness at which AF_M_ assumes a maximum. While our experimental investigation was limited to a small number of compositions, the experimental data mirror the predicted trends, although the experimentally determined AF_E_ values are slightly lower than the predicted AF_M_ values. Nevertheless, the highest AF_E_ value is indeed observed for the thickness combination predicted to yield this maximum, i.e., *l*_PVA_ = 200 μm and *l*_PETG_ = 30 μm (Figure 6, Table S1). Deviating from this optimal geometry by increasing the thickness of either the PETG layer (Figure 6a) or the PVA layer (Figure 6b) leads to a decrease in AF_M_ and AF_E_. We also investigated **PVA**_**100**_ **-PETG**_**200**_ to assess the predictive accuracy of the model at low AF_M_ values. While the model predicts an AF_M_ of 3.5, the experimental AF_E_ is 1.9 ± 0.2, again somewhat lower than the prediction (Table S1). We speculate that the difference observed between AF_M_ and AF_E_ (Table S1) may arise from the presence of the thin (∼15 μm, Table 1) SEBS-MA adhesion layer, which the model does not take into account (Figure 2). In support of this interpretation, the AF_E_ value measured for PVA–PETG bilayer membranes fabricated without the SEBS-MA adhesive interlayer showed improved agreement with the modeled AF_M_ (Figure S9), although the resulting membranes exhibited delamination following the permeability test, suggesting that the resulting AF_E_ value should be interpreted with caution. The fact that the difference between AF_M_ and AF_E_ is more pronounced for thinner PVA layers (*l*_PVA_ = 100 and 200 μm) is consistent with the fact that the relative contribution of the SEBS-MA layer is more significant when the moisture-sensitive PVA layer is thin (Table 1). For thicker PVA layers (*l*_PVA_ = 400 μm), the contribution of the SEBS-MA becomes negligible, which is consistent with the good agreement between AF_M_ and AF_E_ observed for the **PVA**_**400**_ **-PETG**_**30**_ membranes (Figure 6b, Table S1).

## Conclusions

In summary, we demonstrated that dense laminated membranes composed of hydrophilic PVA and hydrophobic PETG can be engineered to exhibit pronounced directional water permeation. While simple bilayer assemblies delaminated under humid conditions, the introduction of a reactive adhesive layer provided robust interfacial adhesion. Guided by modeling, membranes with optimized geometries achieved an asymmetry factor of up to 6.7, one of the highest reported for dense membranes. These results highlight the potential of reactive compatibilizers to enable the design of asymmetric transport properties in otherwise incompatible polymer laminates. We note that a high relative humidity (80–100%, Figure 2) is required to plasticize PVA to the extent that its water permeability is substantially increased and high AF values are attained. To achieve high asymmetry factors at lower relative humidity values, future designs could explore alternate hydrophilic polymers with lower plasticization thresholds.

## Supplementary Material









## Data Availability

The raw data underlying the findings of this work, together with the MATLAB script used for the modeling of the transport properties of the PVA–PETG laminated membranes, can be found at 10.5281/zenodo.17055356

## Notes

Text file with additional experimental details, additional pictures, and SEM images of the PVA–PETG laminated membranes (PDF)
Supplementary videos (mp4) S1–S3 show (S1) the delamination of a PVA–PETG bilayer membrane without the SEBS-MA intermediate layer (MP4)
(S2) the **PVA**_**200**_ **-PETG**_**30**_ membrane with a SEBS-MA intermediate layer after exposure to high RH (MP4)
(S3) a tensile test of the **PVA**_**200**_ **-PETG**_**30**_ membrane after conditioning at high RH (MP4)

## References

[1] Kamtsikakis A., Weder C. (2022). Asymmetric Mass Transport through Dense Heterogeneous Polymer Membranes:
Fundamental Principles, Lessons from Nature, and Artificial Systems. Macromol. Rapid Commun..

[2] Rogers C. E., Stannett V., Szwarc M. (1957). Permeability Valves. Permeability
of Gases and Vapors through Composite Membranes. Ind. Eng. Chem..

[3] Hurst H. (1941). Insect Cuticle
as an Asymmetrical Membrane. Nature.

[4] Hurst H. (1948). Asymmetrical
Behaviour of Insect Cuticle in Relation to Water Permeability. Discuss. Faraday Soc..

[5] Rogers C. E. (1965). Transport
through Polymer Membranes with a Gradient of Inhomogeneity. J. polym. sci., C Polym. symp..

[6] Rogers C. E., Sternberg S., Salovey R. (1968). Preparation and Analysis of Asymmetric
Membranes. J. Polym. Sci. A-1 Polym. Chem..

[7] Rogers C. E., Sternberg S. (1971). Transport
through Permselective Membranes. J. Macromol.
Sci. Part B.

[8] Peterlin A., Williams J. L. (1971). Influence of Compacting
Pressure on the Permeability
of Vectorized Membranes for Liquids. J. Appl.
Polym. Sci..

[9] Peterlin A. (1971). Vapor and
Gas Permeability of Asymmetric Membranes. J.
Appl. Polym. Sci..

[10] Peterlin A. (1972). Asymmetric
Permeability of Vectorized Membranes. Kolloid-Z.u.Z.Polymere.

[11] Peterlin A., Olf H. G. (1972). Steady Flow through
Asymmetric Membranes. J. Macromol. Sci. B.

[12] Petropoulos J. H. (1973). Flow Reversal
Effects in a Simple Laminated Membrane Diffusion System. J. Polym. Sci. Polym. Phys. Ed..

[13] Petropoulos J. H. (1974). “Directional”
Membrane Permeability in Polymer–Vapor Systems. J. Polym. Sci. Polym. Phys. Ed..

[14] Yamanaka T. (1993). Optimization
of Asymmetric Permeation in Heterogeneous Composite Membranes. J. Polym. Sci. B Polym. Phys..

[15] Si Y., Shi S., Dong Z., Wu H., Sun F., Yang J., Hu J. (2023). Bioinspired Stable Single-Layer Janus
Fabric with Directional Water/Moisture
Transport Property for Integrated Personal Cooling Management. Adv. Fiber Mater..

[16] Li K., Ju J., Xue Z., Ma J., Feng L., Gao S., Jiang L. (2013). Structured Cone Arrays for Continuous and Effective
Collection of
Micron-Sized Oil Droplets from Water. Nat. Commun..

[17] Zhang Y., Barboiu M. (2015). Dynameric Asymmetric Membranes for Directional Water
Transport. Chem. Commun..

[18] Kamtsikakis A., McBride S., Zoppe J. O., Weder C. (2021). Cellulose Nanofiber
Nanocomposite Pervaporation Membranes for Ethanol Recovery. ACS Appl. Nano Mater..

[19] Qi L., Ou K., Hou Y., Yuan P., Yu W., Li X., Wang B., He J., Cui S., Chen X. (2021). Unidirectional
Water-Transport Antibacterial Trilayered Nanofiber-Based Wound Dressings
Induced by Hydrophilic-Hydrophobic Gradient and Self-Pumping Effects. Mater. Des..

[20] Hirata Y., Marais S., Nguyen Q. T., Cabot C., Sauvage J.-P. (2005). Relationship
between the Gas and Liquid Water Permeabilities and Membrane Structure
in Homogeneous and Pseudo-Bilayer Membranes Based on Partially Hydrolyzed
Poly­(Ethylene-Co-Vinyl Acetate). J. Membr. Sci..

[21] Kamtsikakis A., Baales J., Zeisler-Diehl V. V., Vanhecke D., Zoppe J. O., Schreiber L., Weder C. (2021). Asymmetric Water Transport in Dense
Leaf Cuticles and Cuticle-Inspired Compositionally Graded Membranes. Nat. Commun..

[22] Kamtsikakis A., Delepierre G., Weder C. (2021). Cellulose Nanocrystals as a Tunable
Nanomaterial for Pervaporation Membranes with Asymmetric Transport
Properties. J. Membr. Sci..

[23] Baloyi R. B., Sithole B. B., Chunilall V. (2024). Physicochemical Properties of Cellulose
Nanocrystals Extracted from Postconsumer Polyester/Cotton-Blended
Fabrics and Their Effects on PVA Composite Films. Polymers.

[24] Mariano M., El Kissi N., Dufresne A. (2014). Cellulose
Nanocrystals and Related
Nanocomposites: Review of Some Properties and Challenges. J. Polym. Sci., Part B: Polym. Phys..

[25] Grillo L., Weder C. (2024). Switchable Asymmetric Water Transport in Dense Nanocomposite Membranes. ACS Appl. Polym. Mater..

[26] Choudalakis G., Gotsis A. D. (2009). Permeability of Polymer/Clay Nanocomposites: A Review. Eur. Polym. J..

[27] Siracusa V. (2012). Food Packaging
Permeability Behaviour: A Report. Int. J. Polym.
Sci..

[28] Alexander S. L. M., Korley L. T. J. (2017). Tunable Hygromorphism:
Structural Implications of Low
Molecular Weight Gels and Electrospun Nanofibers in Bilayer Composites. Soft Matter.

[29] Abdullah Z. W., Dong Y., Han N., Liu S. (2019). Water and Gas Barrier
Properties of Polyvinyl Alcohol (PVA)/Starch (ST)/Glycerol (GL)/Halloysite
Nanotube (HNT) Bionanocomposite Films: Experimental Characterisation
and Modelling Approach. Compos. Part B: Eng..

[30] Mo C., Yuan W., Lei W., Shijiu Y. (2014). Effects of Temperature
and Humidity on the Barrier Properties of Biaxially-Oriented Polypropylene
and Polyvinyl Alcohol Films. J. Appl. Packag.
Res..

[31] Hu H., Zhang X., He Y., Guo Z., Zhang J., Song Y. (2013). Combined Effect of Relative Humidity
and Temperature on Dynamic Viscoelastic
Properties and Glass Transition of Poly­(Vinyl Alcohol). J. Appl. Polym. Sci..

[32] Konidari M. V., Papadokostaki K. G., Sanopoulou M. (2011). Moisture-Induced Effects on the Tensile
Mechanical Properties and Glass-Transition Temperature of Poly­(Vinyl
Alcohol) Films. J. Appl. Polym. Sci..

[33] Hauser P. M., McLaren A. D. (1948). Permeation through
and Sorption of Water Vapor by High
Polymers. Ind. Eng. Chem..

[34] Matsuno I., Takizawa A., Kinoshita T., Tsujita Y. (1987). Water Vapor Permeability
through Laminated Membrane Composed of Hydrophilic and Hydrophobic
Layers. Sen-i Gakkaishi.

[35] Xianda Y., Anlai W., Suqin C. (1987). Water-Vapor Permeability
of Polyvinyl
Alcohol Films. Desalination.

[36] Grillo L., Weder C. (2025). Directional Water Transport in Laminated
Polymer. J. Membr. Sci..

[37] Blom M., van Putten R.-J., van der Maas K., Wang B., Klink G. P. M. v., Gruter G.-J. M. (2024). Terephthalate Copolyesters Based on 2,3-Butanediol
and Ethylene Glycol and Their Properties. Polymers.

[38] Lira M. C. D. A., De Sousa Filho V. A., Da Cunha R. B., De Araújo J. M., Agrawal P., Brito G. D. F., Mélo T. J. A. D. (2025). 4D
Printing Behavior of PETG/SEBS Blends: A Comparative Study of Reactive
and Non-Reactive SEBS with Varied Styrene Content. Polymer.

[39] Silva R., Muniz E. C., Rubira A. F. (2008). Multiple
Hydrophilic Polymer Ultra-Thin
Layers Covalently Anchored to Polyethylene Films. Polymer.

[40] Andre J. S., Li B., Chen X., Paradkar R., Walther B., Feng C., Tucker C., Mohler C., Chen Z. (2021). Interfacial Reaction
of a Maleic Anhydride Grafted Polyolefin with Ethylene Vinyl Alcohol
Copolymer at the Buried Solid/Solid Interface. Polymer.

[41] Shirole A., Sapkota J., Foster E. J., Weder C. (2016). Shape Memory Composites
Based on Electrospun Poly­(Vinyl Alcohol) Fibers and a Thermoplastic
Polyether Block Amide Elastomer. ACS Appl. Mater.
Interfaces.

[42] Greenspan L. (1977). Humidity Fixed
Points of Binary Saturated Aqueous Solutions. J. Res. Natl. Bur. Stan. Sect. A.

[43] ASTM International. ASTM E96/E96M-16 , Standard Test Methods for Water Vapor Transmission of Materials; advancing standards transforming markets 2016. .

[44] Mak-iad C., Bertossi L., Formon G. J. M., Weder C. (2025). Healable Glassy Metallosupramolecular
Polymers. ACS Macro Lett..

