# Supplementary material for: Highly Asymmetric Water Permeation in Dense Laminated Membranes
Source: ACS Appl Polym Mater. 2026 Jan 8;8(2):1107–16. doi: 10.1021/acsapm.5c03886 (PMC12836321; doi:10.1021/acsapm.5c03886)
Supplement: Supplementary file 1 [file ap5c03886_si_001.pdf]

Supporting Information

Highly Asymmetric Water Permeation in Dense Laminated Membranes

*Luca Grillo, Christoph Weder**

*christoph.weder@unifr.ch

Adolphe Merkle Institute, University of Fribourg, Chemin des Verdiers 4, 1700 Fribourg, Switzerland

Supporting Notes

Supporting Note 1: Water Permeability Measurements (ASTM E96)

The normative ASTM E96[1] for the evaluation of water vapor transmission of materials was used to measure the water permeability of the reference PVA and PETG films and the PVA-PETG laminated membranes. The schematic representation of the dry cup and wet cup methods described in the normative is shown in **Figure S1**. The membranes were cut into circles of 6.5 cm in diameter, and the film thickness was calculated with a micrometer (IP 65, Mitutoyo), taking the average of the values measured at 10 random spots per sample. The membranes were conditioned at the test conditions for at least 2 days before mounting them on aluminum cups containing $CaCl_2$ (dry cup method, **Figure S1a**) or DI water (wet cup method, **Figure S1b**). The cups were placed in a ventilated incubator kept at $T = 25$ °C. For the dry cup method, $CaCl_2$ was dried at 200°C before use to ensure 0% RH in the receiver compartment inside the cups (assumption $RH_R = 0\%$),[2] while different values of relative humidity of the donor compartment outside the cups (RH_D) were generated by placing saturated salt solutions in the incubator. More specifically, water solutions of $Mg(NO_3)_2$ ($RH_D = \sim 55\%$), NaCl ($RH_D = \sim 75\%$), KCl ($RH_D = \sim 85\%$), or K_2SO_4 ($RH_D = \sim 95\%$)[3] were used to reach RH_D values of 55, 70, 80, 85, 90, and 95%. During the test, the RH inside the incubator was monitored using a humidity thermometer (Fisherbrand™ Traceable™ humidity thermometer). The values of RH_D reported in **Figure 1** of the main manuscript are the average values measured during the water permeability test. The variations of the average with respect to the target values of RH_D are within the accuracy of the monitoring device (±4%). When the wet cup method was employed, ca. 500 g of $CaCl_2$ were introduced in the incubator to keep dry conditions in the receiver compartment (assumption $RH_R = 0\%$).[2] The cups were removed from

the incubator at regular time intervals ($1 < \Delta t < 48$h) and weighed to evaluate the water vapor transmission rate (*WVTR*), whose definition is reported in **Equation S1**:

$$WVTR = \frac{g}{A \cdot t} \tag{S1}$$

Where g is the change in mass of the assembly, A is the transport area, and t is the time.

At least 5 data points over at least 24 hours were collected to perform the linear regression to obtain the *WVTR*. **Equation S2** was used to calculate the water permeability (*WP*):

$$WP = \frac{WVTR \times l}{\Delta p} \tag{S2}$$

Where l and Δp are the average film thickness and water vapor pressure gradient applied to the membrane, respectively. The gradient Δp can be expressed in terms of the difference in relative humidity generated between the donor (RH_D) and the receiver (RH_R) compartments using **Equation S3**.

$$\Delta p = (RH_D - RH_R)p_{sat} \tag{S3}$$

Where p_{sat} is the saturated water vapor pressure (p_{sat}= 3162 Pa at T = 25 °C). The water permeability measurements through the **PVA_x-$PETG_y$** membranes were conducted in two directions, i.e., each side of the laminated membranes facing the donor side once, and the reported values were the mean and standard deviation of n = 3 membranes.

The asymmetric transport was expressed according to the definition of the asymmetry factor AF_E (the subscript E is used to distinguish the experimentally determined values from the modeling results), which is reported in **Equation S4.**

$$AF_E = \frac{WP_{PVA \to PETG}}{WP_{PETG \to PVA}} \tag{S4}$$

Where $WP_{PVA \to PETG}$ and $WP_{PETG \to PVA}$ correspond to the water permeability of the PVA-PETG laminated membrane evaluated in the direction from the PVA layer to the PETG layer and vice versa, respectively.

Supporting Note 2: Modeled Asymmetry Factor AF_M

Based on Petropoulos' theoretical study,[4] we define the modeled asymmetry factor AF_M as the ratio of the normalized permeation rates in the two directions of transport, PVA→PETG and PETG→PVA, as shown in Equation S5.

$$AF_M = \frac{\hat{J}_{PVA \to PETG}}{\hat{J}_{PETG \to PVA}} \quad \textbf{(S5)}$$

The normalized permeation rate $\hat{J}$ can be expressed with **Equation S6,** where l is the thickness of a generic membrane subjected to a gradient in relative humidity (RH_D - RH_R).

$$\hat{J} = \frac{1}{l} \int_{RH_R}^{RH_D} WP(RH)\, dRH \quad \textbf{(S6)}$$

If we express the normalized permeation rate as the mass flow through the PETG layer, the normalized permeation rates of **Equation S5** can be expressed by **Equations S7** and **S8** depending on the transport direction:

PVA→PETG
$$\hat{J}_{PVA \to PETG} = \frac{1}{l_{PETG}} \int_{0}^{RH'_m} WP_{PETG} \quad \textbf{(S7)}$$

PETG→PVA
$$\hat{J}_{PETG \to PVA} = \frac{1}{l_{PETG}} \int_{RH^*_m}^{RH_D} WP_{PETG} \quad \textbf{(S8)}$$

The extremes of integration refer to the RH to which the PETG layer is subjected according to the scheme in **Figure 2** of the main manuscript.

By replacing the normalized permeation rates in **Equation S5**, the asymmetry factor from the model AF_M can be calculated with **Equation S9**.

$$AF_M = \frac{\int_0^{RH'_m} WP_{PETG}}{\int_{RH^*_m}^{RH_D} WP_{PETG}} \quad \textbf{(S9)}$$

Since WP_{PETG} is constant, it cancels out from **Equation S9,** and AF_M is given by the ratio shown in **Equation S10**.

$$AF_M = \frac{RH'_m}{RH_D - RH^*_m} \qquad \textbf{(S10)}$$

Where RH_m' and RH_m* are the intermediate values of RH at the interface between the PVA and PETG layer in the PVA→PETG and PETG→PVA transport directions, respectively (**Figure 2** of the main manuscript).

Supporting Figures

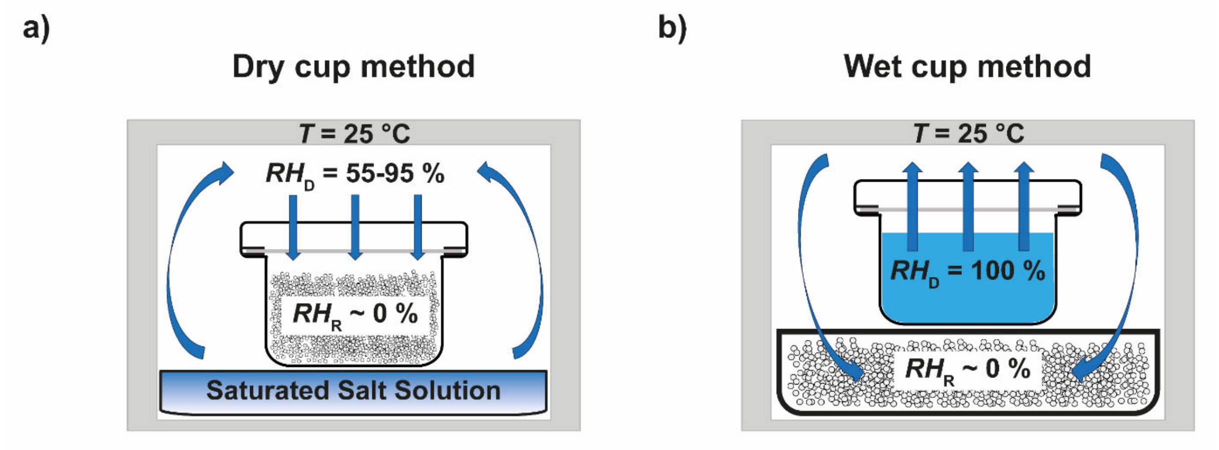

Figure S1: Schematic representation of the (a) dry and (b) wet cup methods described in the normative ASTM E96 that were used to measure the water permeability (*WP*) of the various membranes at 25 °C.

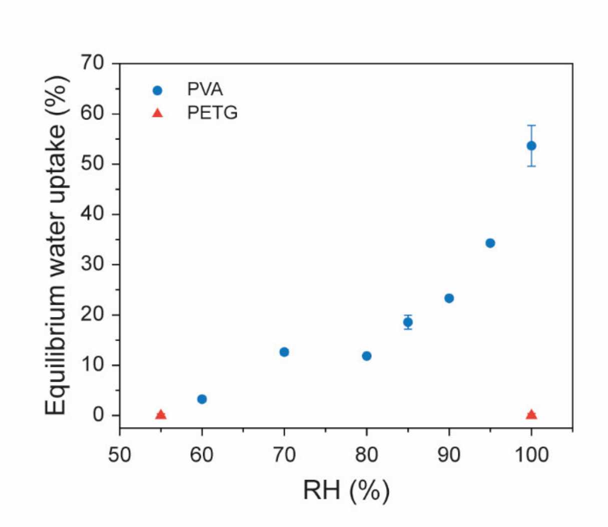

Figure S2: Equilibrium water uptake of neat PVA and PETG reference films at different relative humidity (*RH*). The reported values are the mean and the standard deviation of $n = 3$ measurements for each film.

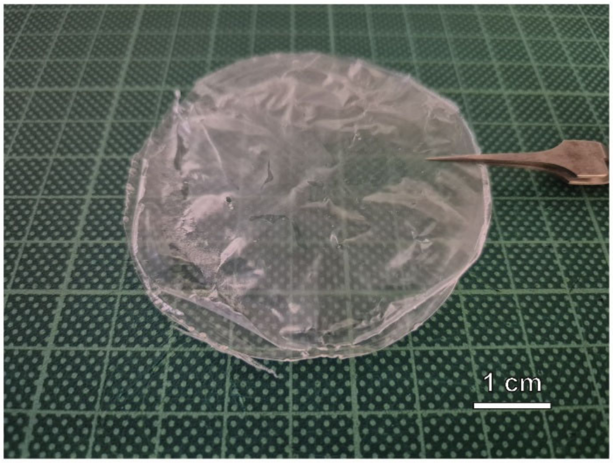

Figure S3: Picture showing the delamination between PVA (200 µm) and PETG (30 µm) layers of a PVA-PETG membrane prepared without the SEBS-MA intermediate layer. The membrane was exposed to *RH* ~95% overnight.

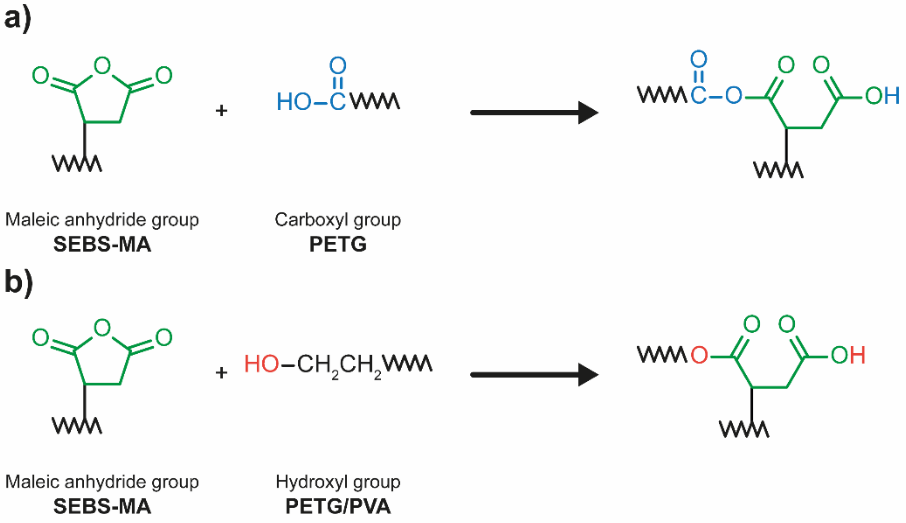

Figure S4: Schematic representation of the possible reactions between the maleic anhydride group present in SEBS-MA and (a) the carboxyl end groups present in PETG and (b) the hydroxyl groups present as end groups in PETG and side groups in PVA. The possible occurrence of these reactions is supported by previously reported studies in both bulk[5] and interfacial systems.[6,7]

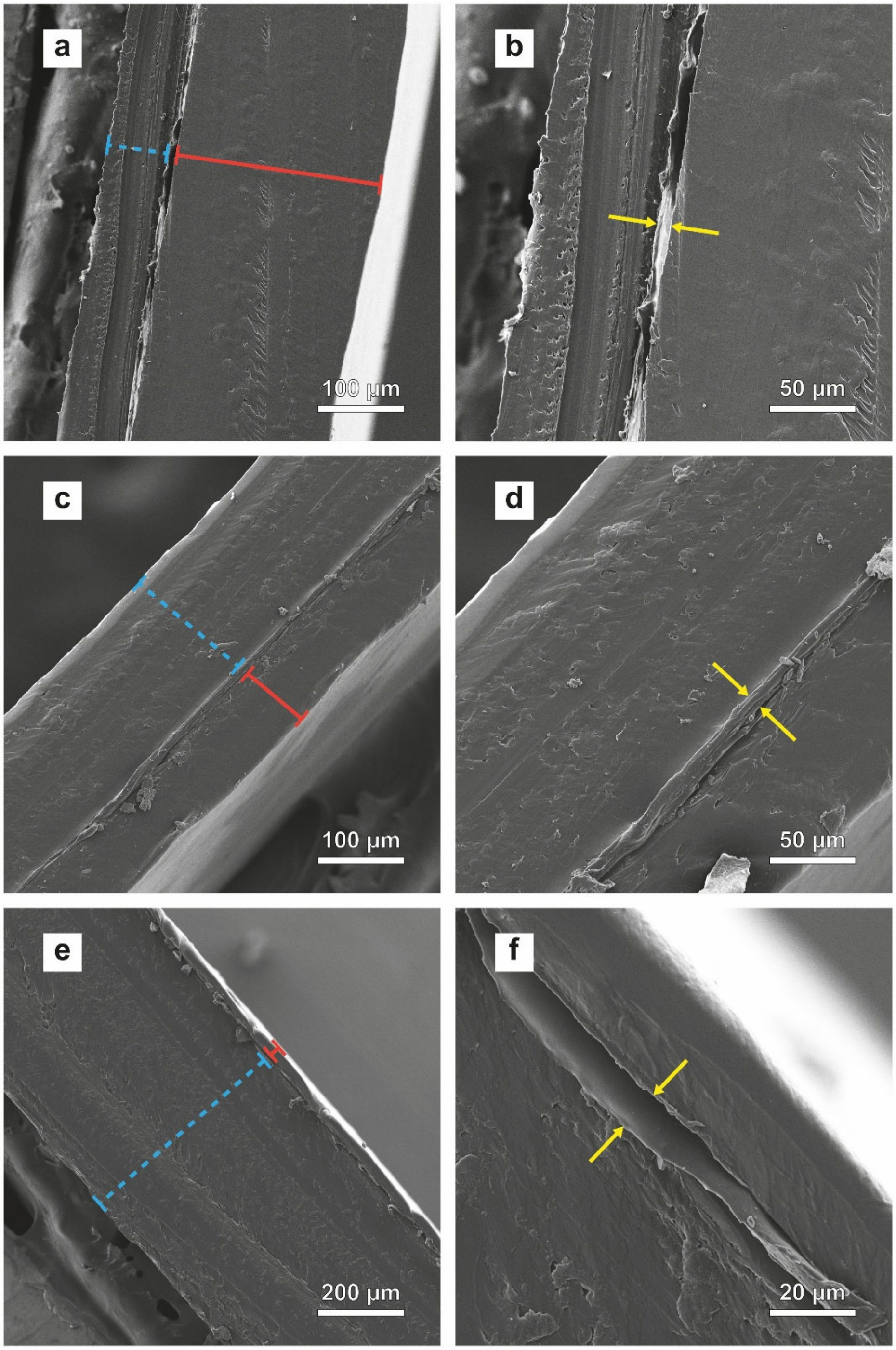

Figure S5: SEM images of the cross-sections of the (a, b) **PVA_{100}-$PETG_{200}$**, (c, d) **PVA_{200}-$PETG_{70}$**, and (e, f) **PVA_{400}-$PETG_{30}$** membranes. The dashed blue lines mark the PVA layers, the solid red lines the PETG layers, and the yellow arrows the SEBS-MA intermediate layers.

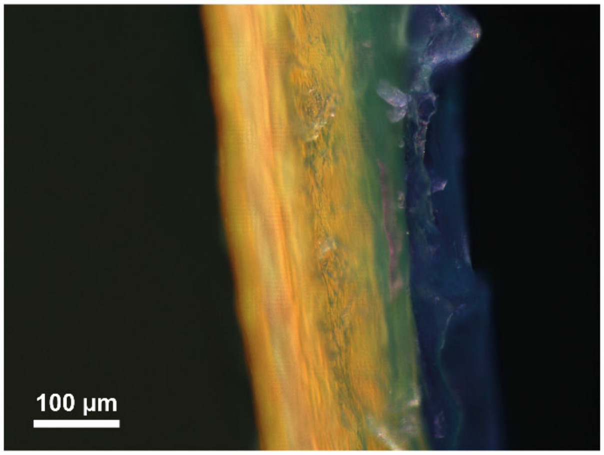

Figure S6: Fluorescence microscopy image of a dye-labeled **PVA_{200}-$PETG_{30}$** membrane. The PVA layer was labeled with rhodamine (red), the SEBS-MA adhesion layer was labeled with fluorescein (green), and the PETG layer was labeled with indigo (blue). The image was acquired upon excitation with UV light (365 nm).

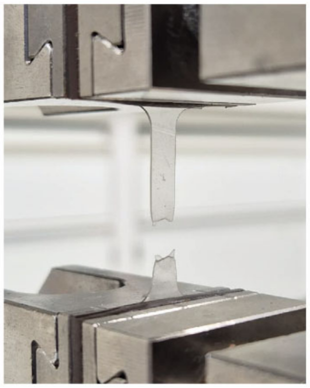

Figure S7: Picture capturing the failure mechanism of a **PVA_{200}-$PETG_{30}$** membrane after tensile testing under dry conditions. The image shows that the membrane breaks homogeneously, without signs of delamination between the PVA and PETG layers.

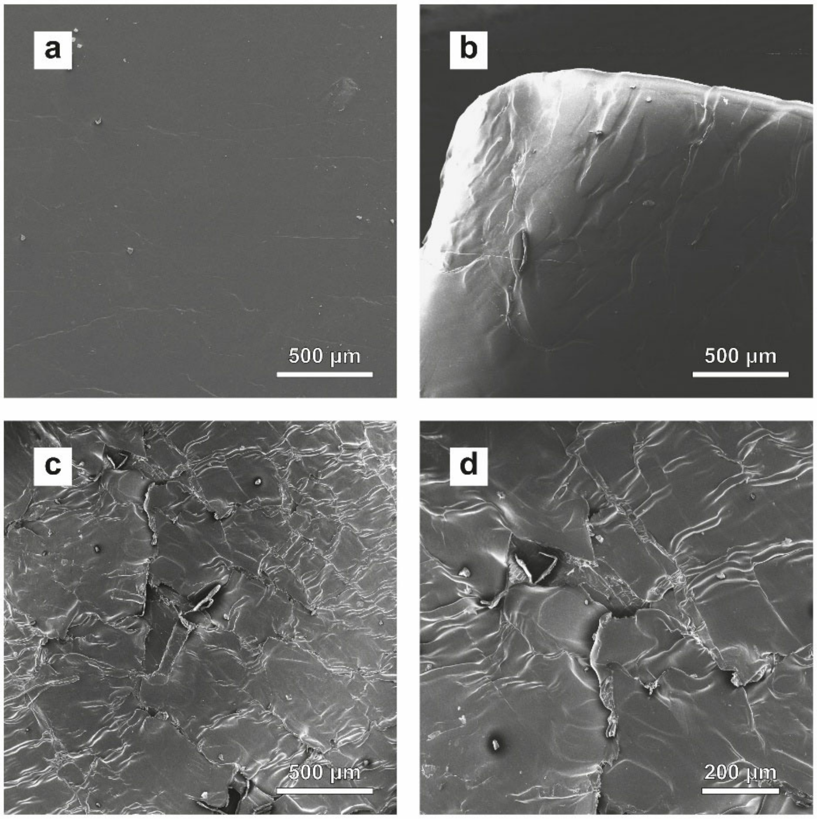

Figure S8: SEM images of the PETG sides of the **PVA_{200}-$PETG_{30}$** membranes (a) before tensile testing and after testing the samples under (b) dry and (c, d) humid conditions (conditioning the samples at *RH* ~ 95% for one week before the mechanical testing).

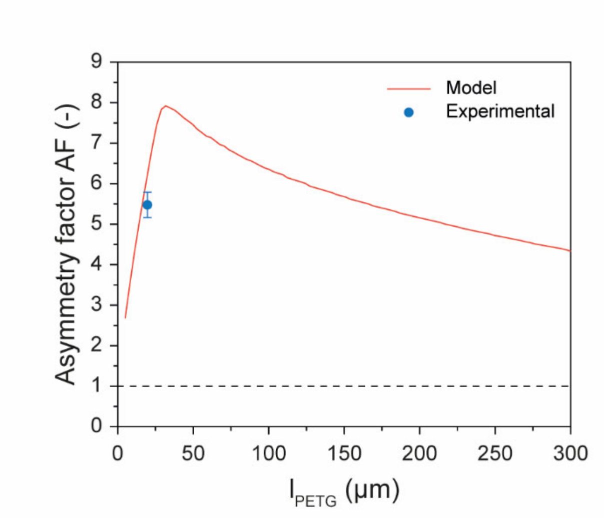

Figure S9: Comparison between the asymmetry factor of laminated PVA-PETG membranes predicted by the model (AF_M) and the experimentally determined values (AF_E) at RH_D = 100% and RH_R = 0% for different thickness combinations. The experimental value was measured in PVA-PETG bilayer membranes prepared without the SEBS-MA interlayer, using the wet cup method, and represents the mean ± s.d. of n = 3 membranes.

Supporting Tables

Table S1: Comparison between the experimental values of the asymmetry factor (AF_E) and the predicted values estimated with the model (AF_M) for the **PVA_x-$PETG_y$** laminated membranes.

Membrane	AF_E [-]	AF_M [-]
**PVA_{100}-$PETG_{200}$**	1.9 ± 0.2	3.5
**PVA_{200}-$PETG_{70}$**	5.5 ± 0.5	7.0
**PVA_{200}-$PETG_{30}$**	6.7 ± 0.5	7.9
**PVA_{400}-$PETG_{30}$**	5.0 ± 0.9	5.4

Supporting References

(1) ASTM International. ASTM E96/E96M-16, Standard Test Methods for Water Vapor Transmission of Materials, 2016. https://doi.org/10.1520/E0096_E0096M-16.

(2) Kamtsikakis, A.; Baales, J.; Zeisler-Diehl, V. V.; Vanhecke, D.; Zoppe, J. O.; Schreiber, L.; Weder, C. Asymmetric Water Transport in Dense Leaf Cuticles and Cuticle-Inspired Compositionally Graded Membranes. *Nat. Commun.* **2021**, *12* (1), 1267. https://doi.org/10.1038/s41467-021-21500-0.

(3) Greenspan, L. Humidity Fixed Points of Binary Saturated Aqueous Solutions. *J. Res. Natl. Bur. Stan. Sect. A.* **1977**, *81A* (1), 89. https://doi.org/10.6028/jres.081A.011.

(4) Petropoulos, J. H. "Directional" Membrane Permeability in Polymer–Vapor Systems. *J. Polym. Sci. Polym. Phys. Ed.* **1974**, *12* (1), 35–49. https://doi.org/10.1002/pol.1974.180120104.

(5) Lira, M. C. D. A.; De Sousa Filho, V. A.; Da Cunha, R. B.; De Araújo, J. M.; Agrawal, P.; Brito, G. D. F.; Mélo, T. J. A. D. 4D Printing Behavior of PETG/SEBS Blends: A Comparative Study of Reactive and Non-Reactive SEBS with Varied Styrene Content. *Polymer* **2025**, *319*, 128059. https://doi.org/10.1016/j.polymer.2025.128059.

(6) Silva, R.; Muniz, E. C.; Rubira, A. F. Multiple Hydrophilic Polymer Ultra-Thin Layers Covalently Anchored to Polyethylene Films. *Polymer* **2008**, *49* (19), 4066–4075. https://doi.org/10.1016/j.polymer.2008.07.051.

(7) Andre, J. S.; Li, B.; Chen, X.; Paradkar, R.; Walther, B.; Feng, C.; Tucker, C.; Mohler, C.; Chen, Z. Interfacial Reaction of a Maleic Anhydride Grafted Polyolefin with Ethylene Vinyl Alcohol Copolymer at the Buried Solid/Solid Interface. *Polymer* **2021**, *212*, 123141. https://doi.org/10.1016/j.polymer.2020.123141.
